# Synergistic Effect of Composite Chitosan/Phenylalanine Edible Coating on the Postharvest Bioactive Composition of Mandarin Fruit

**DOI:** 10.1002/fsn3.71239

**Published:** 2025-12-12

**Authors:** Slaven Jurić, Marko Vuković, Marija Sigurnjak Bureš, Katarina Sopko Stracenski, Francesco Donsì, Luna Maslov Bandić

**Affiliations:** ^1^ Department of Chemistry University of Zagreb Faculty of Agriculture Zagreb Croatia; ^2^ Department of Pomology University of Zagreb Faculty of Agriculture Zagreb Croatia; ^3^ Department of Industrial Engineering University of Salerno Fisciano Italy

**Keywords:** bioactive compounds, chitosan, edible coatings, mandarin fruit, phenylalanine

## Abstract

This study investigates the effects of Chitosan and Chitosan/Phenylalanine (Phe) edible coatings on the postharvest quality of mandarins during shelf life at room temperature (21°C) and during cold storage (5°C). Mandarins were treated with Chitosan, Phe, and a combination of Chitosan/Phe, with untreated mandarins serving as a control. Several key quality parameters, including total polyphenolic content (TPC), total flavonoid content (TFC), antioxidant activity (DPPH and ABTS assays), organic acids, and aroma compounds were analyzed at multiple time points. Chitosan and Chitosan/Phe improved glossiness and effectively preserved mandarins by lowering the decay rate and modulating bioactive composition. Chitosan and Chitosan/Phe treatments showed similar outcomes on bioactive composition but Chitosan/Phe treatment was found to be favored. Chitosan/Phe is the most effective treatment, extending mandarin shelf life by maintaining superior nutritional, antioxidant, and sensory quality up to 12 days at room temperature and 35 days under cold storage. Chitosan/Phe coating after 12 days of shelf life significantly reduced weight loss in mandarins, while Chitosan treatment was comparable to Phe and control samples. Additionally, relative to the control, Chitosan/Phe had the most pronounced effect on TPC after 35 days of cold storage with an increase of up to 78.98%, while Chitosan coating resulted in a 40.93% increase, respectively. Chitosan/Phe coating effectively increased TFC (narirutin and hesperidin content) after 12 days of shelf life by 42.79% compared to the control. Organic acids, especially oxalic and citric acids, were positively affected by the Chitosan and Chitosan/Phe coatings, contributing to delayed senescence. Furthermore, the Chitosan/Phe treatment was associated with enhanced volatile compound retention, improving the mandarin aroma profile, particularly in cold storage. Overall, both Chitosan and Chitosan/Phe treatments improved nutritional and sensory quality while reducing fruit decay, but Chitosan/Phe was able to reduce fruit weight loss after shelf life making this combination a more effective solution to extend the shelf life and marketability of mandarins.

## Graphical abstract

Chitosan/Phenylalanine (Chitosan/Phe) composite coating markedly enhanced the postharvest stability and biochemical integrity of mandarin fruit. This treatment effectively extended shelf life to 12 days at ambient conditions and 35 days under cold storage, while significantly increasing polyphenolic and flavonoid content and sustaining antioxidant activity. Moreover, Chitosan/Phe coatings improved volatile retention, surface gloss, and overall sensory quality.

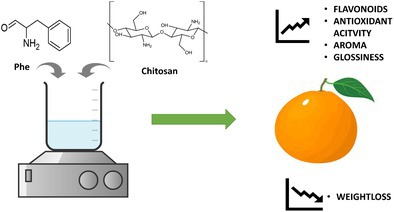

## 1 Introduction

Mandarin fruits have gained significant global popularity as a result of their delicate taste, distinctive flavor and nutritional quality (Feng et al. 2018). Beyond their delicious taste, mandarins also offer a range of health benefits, such as antioxidant, anticancer, anti‐inflammation, anti‐hyperlipidemic and anti‐diabetic properties (Wang et al. 2017). These benefits are attributed to the presence of phytochemicals including organic acids, sugars, and amino acids, alongside secondary metabolites such as carotenoids, polyphenols, flavonoids, phenolic acids, and limonoids (Tietel et al. 2020). However, due to their perishable nature and being non‐climacteric, mandarins cannot be transported or stored for an extended period (Rokaya et al. 2016). The Unshiu mandarin (*Citrus unshiu* Marc.) is the major Citrus crop in Croatia, which in 2022, together with clementines, were cultivated on 2040 ha with a total yield of 41,950 t (FAOSTAT 2024). This crop holds particular importance for Croatia, as much of its production is destined for export.

Reducing fruit loss, minimizing weight reduction, enhancing fruit quality, and improving profitability can be achieved through appropriate storage and postharvest treatments. In recent times, there has been a growing focus on the utilization of natural and safe ingredients to improve fruit quality and extend shelf life, which has positively impacted fruit marketing. Edible coatings, defined as thin layers of biodegradable and edible components that encapsulate fruit, have gained attention for their ability to protect against mechanical, physical, chemical, and microbial damage while also providing a desirable appearance (Saidi et al. 2021). These coatings create a modified micro‐environment around the fruit, regulating water vapor, carbon dioxide, and oxygen exchange, thereby extending shelf life without affecting fruit safety and quality parameters (Hassan et al. 2018).

The primary constituents of edible coatings are polysaccharides (e.g., chitosan, alginate, cellulose, pectin, and starch), proteins (e.g., gelatin, zein, soy protein, and casein), lipids (e.g., beeswax, carnauba wax, and vegetable oils), and composites thereof (Kumar et al. 2023, 2024). Chitosan, a natural polysaccharide, is widely used in postharvest edible coatings for various fruits and vegetables (Arnon‐Rips and Poverenov 2018). Chitosan possesses noteworthy characteristics, such as inhibiting fungal proliferation, delaying water loss, and preventing browning (Obianom et al. 2019). Thus, the development of chitosan‐based preservation methods is expanding rapidly, with various natural additives being incorporated to enhance its performance. For example, chitosan coating with tea polyphenols has been successfully applied to broccoli (Fang et al. 2022), while pomelo extract‐enriched chitosan has been applied to coat lychee (Yang et al. 2023), improving shelf life and marketability of fruits, while enhancing sensory quality. Jiao et al. (2019) applied chitosan coating with chlorogenic acid on peaches, maintaining fruit quality, while chitosan with ascorbic acid (Saleem et al. 2021) and apple peel polyphenols (Riaz et al. 2021) were applied to strawberries, improving shelf life, reducing weight loss and decay, and preserving high levels of polyphenols. Additionally, chitosan coatings with ginger extract have been applied to walnuts and managed, reducing oxidation, and growth of *Aspergillus flavus* (Shaukat et al. 2023).

One promising additive to boost the performance of the edible coatings for mandarins is the amino acid phenylalanine (Phe). Phe has gained significant attention for its potential to improve the nutritional value of horticulture crops. Phe is capable of exerting both direct and indirect effects on the physiological activities of plants, and plays a key role in numerous metabolic reactions that occur within the plant, via the phenylpropanoid pathway. Phe, as one of the essential amino acids, plays a critical role in the biosynthesis of antioxidants and aromatic chemicals (Aghaei et al. 2019). The phenylpropanoid pathway, initiated by Phe catalyzed by phenylalanine ammonia‐lyase (PAL), leads to the production of various metabolites, such as phenolics, flavonoids, anthocyanins, and lignins (Patel, Fanyuk, et al. 2023; Patel, Maurer, et al. 2023). It has been reported that exogenous Phe triggers different metabolic pathways and antioxidant enzyme activity (Patel, Maurer, et al. 2023). Both preharvest and postharvest treatments with Phe have been found to stimulate fruit defense responses, inhibiting different fungal pathogens and reducing postharvest decay (Saidi et al. 2021; Kumar Patel et al. 2020).

This work investigates the application of Phe, chitosan coating and chitosan coating with the addition of Phe on “Owari satsuma” mandarin fruit. The objective was to assess the effects of these postharvest treatments on the metabolism of mandarins during cold storage and shelf life. Specifically, this work evaluates changes in pomological parameters and bioactive compounds such as polyphenols, flavonoids, synephrine, proteins, and organic acids, as well as antioxidant activity, which is pivotal in determining the nutritional quality and shelf life of the fruit. Understanding the impact of these natural treatments on the biochemical composition of mandarins can help develop effective postharvest methods to improve fruit quality, and meet consumer expectations for healthier, longer‐lasting produce.

## 2 Materials and Methods

### 2.1 Plant Material

Mandarins “Owari satsuma” (*Citrus unshiu* Marcovitch), were obtained from one of the commercial orchards (Mandarinko d.o.o.) located at Opuzen, Neretva valley, Croatia (Latitude: 43.0176, Longitude: 17.5623; 43°1′3″ North, 17°33′44″ East). The fruits were harvested (on the 4th of December, 2023) at optimal maturity and immediately delivered to the laboratory of the Department of Chemistry, University of Zagreb Faculty of Agriculture for further processing. Harvest maturity parameters of the mandarin fruit were recorded for 30 randomly selected mandarin fruits. Average fruit weight was 85.25 ± 17.76 g with total soluble solids of 11.10% ± 0.98% and titratable acidity of 0.83% ± 0.12% (as citric acid).

### 2.2 Chemicals

High molecular weight chitosan (CAS Number: 9012‐76‐4, molecular weight: 310000–375,000 Da; 800–2000 cP, 1 wt% in 1% acetic acid (25°C, Brookfield)) was purchased from Sigma‐Aldrich (USA). L‐Phenylalanine, 98.5%–101.0%, was sourced from Thermo Scientific Chemicals (USA). All of the analytical standards and chemicals used in the chemical analysis were purchased from Sigma‐Aldrich (USA). All other chemicals were of analytical grade and used as received without further purification.

### 2.3 Preparation of Dipping Solutions

A volume of 5 L of 2% (w/v) chitosan was prepared by dissolving it in a sterile 2% (w/v) citric acid solution and left overnight under constant stirring at room temperature (20°C ± 2°C). Glycerol was added as a plasticizer (2% v/v). For the Chitosan/Phe treatment, Phe was additionally dissolved directly in the chitosan solution before adjusting the volume to the final concentration. Based on previous research, the optimal Phe concentration for postharvest application on mandarins was determined to be 6 mM (Kumar Patel et al. 2020). A Phe solution of the same concentration, prepared in distilled water, was used as a positive control. The solutions were transferred in separate baths for dipping.

Before post‐harvest treatments, mandarins were washed with cold tap water and air‐dried. Untreated mandarin fruits served as control samples. For coating treatments, the mandarins were submerged in dipping solutions for 3 min and then allowed to dry in a well‐ventilated room equipped with fans to ensure good airflow (approx. for 6 h). For each treatment, 3 sets of 30 fruits were randomly selected for room temperature storage, and another 3 sets of 30 fruits for cold storage (at 5°C and 85% relative humidity). Samples were collected on specified days: 15 fruits per replication were taken for analysis. Mandarins kept at room temperature (20°C ± 2°C, 50% ± 10% relative humidity) for common store were sampled on the 5th, 9th and 12th days, while those at cold storage were sampled on the 7th and 35th days. The extended cold storage period was used to assess the long‐term efficacy of the edible coatings.

### 2.4 Fruit Weight Loss and Decay Rate

Fruit weight loss and decay rate were measured before treatment and at each sampling point for all remaining mandarins.

The fruit weight loss (%) was determined according to Equation (1):

(1) $$\text{Weight loss}\;\left(\%\right)=\frac{a-b}{a}\times 100$$

where *a* is the initial weight at the start of the storage, and *b* is the weight on the inspection date or the final weight.

The decay rate was calculated using the following Formula (2):

(2) $$\text{Decay rate}\;\left(\%\right)=\frac{\text{total number of decayed fruits}}{\text{total number of examined fruits}}\times 100$$

### 2.5 Visual Observation Over Time and Fruit Gloss Sensory Analysis

Twenty randomly selected (5 per treatment) mandarins were kept at room temperature in a fume hood and photographed on each inspection date. Fruit gloss was rated on a 0–10 scale, where 0 represented no gloss and 10 represented very glossy. For each treatment, five mandarins were randomly selected in three repetitions from their respective batches. Randomized blocks were prepared for evaluation, with six repetitions. A total of twelve participants conducted a subjective evaluation of the treated mandarins using a paired comparison blind test (Arnon et al. 2015).

### 2.6 Preparation of Mandarin Fruit Juice for Biochemical Analysis

Ten fruits per treatment, in three repetitions, were used for juice preparation (resulting in 3 juice samples per treatment). The mandarin was weighed, peeled, and blended using a FOSS homogenizer 2094 (Hillerød, Denmark). For each fruit, weight was recorded before and after peeling (to determine the share of pulp %). The resulting homogenized puree was transferred into Falcon tubes (50 mL) and centrifuged at 11,180 × *g* and 4°C, for 10 min. The weights of supernatants and pellets were recorded before and after homogenization and centrifugation, respectively. The supernatant was then filtered using Whatman no. 4 filter paper and used for further analysis, with the juice being diluted as necessary.

### 2.7 Total Soluble Solids and Titratable Acidity

The total soluble solids (TSS) of mandarins were determined using a digital hand refractometer (PAL‐1; Atago, Tokyo, Japan) and expressed in percentage (%). Titratable acidity (TA) was determined via titration method with 0.1 M NaOH and expressed as a % of citric acid. TSS and TA were measured before treatment and at each sampling point for all remaining mandarins thereafter. TSS_TA was evaluated as the ratio of TSS over TA and was calculated by dividing the TSS value by the TA value for each sample.

### 2.8 Determination of Total Protein Content

The total protein content in mandarin juice was determined using the Lowry method (Satpathy et al. 2020). Three reagents were prepared: Reagent A [2% (w/v) Na_2_CO_3_ in 0.1 mol/dm^3^ NaOH], Reagent B [0.5% (w/v) CuSO_4_ x 5H_2_O in 1% (w/v) KNaC_4_H_4_O_6_·4H_2_O] and Reagent C [which consisted of 50 parts of Reagent A and one part of Reagent B]. A volume of 0.2 mL of Folin‐Ciocalteau reagent (1:2 parts of water, v/v) was added to 0.4 mL of juice in a test tube. Finally, reagent C (2 mL) was added and 50 min after the start of the chemical reaction, absorbance was measured at 740 nm against a blank using a UV‐1700 Spectrophotometer (Shimadzu, Kyoto, Japan). A bovine serum albumin standard was used for the calibration curve.

### 2.9 Determination of Total Polyphenolic Content, Total Flavonoids, and Antioxidant Activity (DPPH and ABTS)

The modified Folin Ciocalteu's method (Singleton et al. 1999) was used to determine total polyphenolic content (TPC). Briefly, mandarin juice (100 μL) was mixed with 7.9 mL of distilled water and 500 μL of Folin Ciocalteu's reagent (diluted with distilled water in a 1:2 ratio). A volume of 1.5 mL of 20% (w/v) Na_2_CO_3_ was added to the suspension which was vortexed and left for 2 h to react. Gallic acid was used as a standard. Absorbance was measured at 765 nm, and the data are expressed as mg of gallic acid equivalents per weight of mandarin pulp.

Total flavonoid (TF) content was determined by the modified spectrophotometric method of Ivanova et al. (2010). A volume of 1 mL of mandarin juice was added to a 10 mL volumetric flask containing 4 mL of distilled water. Then, 300 μL of NaNO_2_ (0.5 g/L) solution was added. After 5 min, 300 μL of AlCl_3_ (1 g/L) solution was added and 6 min later, 2 mL of NaOH (1 M) was added to the mixture. The final volume was adjusted to 10 mL with the addition of distilled water. The solution was mixed and the absorbance was measured at 360 nm. Quercetin was used as a standard compound.

The antioxidant potential of mandarin juice was determined using 2,2‐diphenyl‐1‐picrylhydrazyl (DPPH) and 2,2′‐azino‐bis (3‐ethylbenzothiazoline‐6‐sulfonic acid) (ABTS) reagents, according to the known procedures (Brand‐Williams et al. 1995; Re et al. 1999), respectively. For the DPPH method, a volume of 3.9 mL methanolic DPPH solution was added to the test tube containing 100 μL of mandarin juice. The free radical‐scavenging capacity of the sample was determined by measuring the absorbance decrease at 517 nm after 30 min of incubation against the blank sample. For the ABTS method, an amount of 40 μL of mandarin juice was added to 4 mL of the ABTS radical solution in a test tube, and the absorbance readings were taken after exactly 6 min against the appropriate reagent blank instead of the sample. A water‐soluble vitamin E analog Trolox ((±)‐6‐Hydroxy‐2,5,7,8‐tetramethylchromane‐2‐carboxylic acid) was used as the standard compound.

### 2.10 Determination of Narirutin and Hesperidin

Extraction of flavonoids was conducted according to Wang et al. (2008) with minor modifications. A 0.1 g of lyophilized pulp was mixed with 1.5 mL of methanol/dimethylsulfoxide (1:1, v/v) in an Eppendorf tube and mixed in an ultrasonic bath (Elma S 10H Elmasonic, Elma Schmidbauer, Germany) for 10 min at room temperature. After sonication, the samples were centrifuged at 9000 rpm for 10 min at 4°C. The residues were extracted twice with 1.5 mL of methanol/dimethylsulfoxide (1:1, v/v). All supernatants were collected in a volumetric flask (5 mL) and filled up with methanol. The extracts were filtered through 0.45 μm LLG‐RC syringe filters (LLG Gmbh, Grevenbroich, Germany) before use. Extractions were performed in triplicate.

Analysis was carried out using an HPLC (high‐performance liquid chromatography) system (Agilent 1260 Infinity II System, Germany), equipped with an autosampler, quaternary pump, column thermostat and DAD detector. The separation of flavonoids was conducted using an Agilent Poroshell 120 SB‐C18 150 × 4.6 mm 4 μm (Agilent, Palo Alto, CA, USA) at 40°C and 0.8 mL/min. The injected volume was 20 μL. The mobile phase consisted of (A) aqueous 2% formic acid and (B) methanol, with a gradient elution as follows: 0 min, 10% B; 10 min, 20% B; 20 min, 30% B;30 min 40% B; 35 min 40% B; 42 min, 50% B; 52 min, 90% B; 53 min 10% B; 60 min 10% B. Flavonoids were quantified at wavelengths of 280 and 330 nm.

### 2.11 Determination of Synephrine

The concentration of synephrine was determined with the HPLC system equipped with a Phenomenex Luna pentafluorophenyl column, 150 × 3 mm i.d., 5 μm. Juice samples were filtered through a 0.45 μm pore size LLG‐RC syringe filter. The mobile phases were 5 mmol/L ammonium acetate (A) and methanol (B), with isocratic elution of 10% B, and a flow rate of 1 mL/min. Synephrine was quantified at a wavelength of 225 nm. The volume of sample injected was 5 μL, and the column temperature was 20°C. Injections were performed in triplicate.

### 2.12 Determination of Organic Acids

A modified HPLC method was used to determine the content of oxalic, malic, ascorbic, and citric acids in mandarine juices (Nour et al. 2010). Mandarin juices were diluted at 1:50 for citric acid determination and 1:5 for other investigated acids. Diluted juice was filtered by LLG‐RC 0.45 μm syringe filters (LLG Gmbh, Grevenbroich, Germany) before analysis. Agilent 1260 Infinity II System (Agilent, Waldbronn, Germany), equipped with an autosampler, column thermostat, and DAD (diode‐array detector) was used. The separation of organic acids was performed on COSMOSIL C18‐PAQ (250 mm × 4.6 mm i.d., 5 μm) at 40°C. The detection wavelengths were 254 nm for ascorbic acid and 210 nm for other acids. The injected sample volume was 20 μL. The mobile phase was 50 mM phosphate buffer with isocratic elution at a flow rate of 0.7 mL/min for the determination of oxalic, malic, ascorbic, and citric acids.

### 2.13 The Aromatic Profile of Mandarins

Headspace‐solid phase microextraction extraction (HS‐SPME) was used to assess volatile compounds from mandarin fruits. Aroma volatiles were isolated from homogenized fruit segments. To prevent enzymatic deterioration, the fruits were weighed, carefully peeled by hand to prevent the introduction of peel oil into the juice fraction, and then blended for 30 s with an equal weight of 30% aqueous NaCl solution as described (Goldenberg et al. 2015). HS‐SPME was conducted to extract the volatile compounds according to Xiao et al. (2017), with minor modifications. Exactly 9.17 g of prepared sample was mixed with 1.5 g of sodium chloride. The mixture was transferred into a 15 mL headspace bottle with PTFE (PolyTetraFluoroEthylene)/silicone septum (Supelco, Bellefonte, PA, USA) and 15 μL (15.7 mg/L) 2‐octanol (internal standard) was added. Afterward, the bottle was set into a dry block heater (IKA‐Werke GmbH Germany) at a magnetic stirrer (Heidolph GmbH & Co, Germany) with a 50/30 μm divinylbenzene/carboxen/polydimethylsiloxane (DVB/CAR/PDMS) fiber (Supelco, Bellefonte, PA, USA). Fiber was positioned around 1 cm above the surface of the sample. The mixture was heated at 45°C and stirred at 200 rpm for 30 min. Finally, fiber was set into the injector port of the instrument GC to desorb for 5 min at 250°C. The fiber was conditioned for 30 min at 250°C to remove any possible residues. This procedure was repeated for each sample.

Gas Chromatography–Mass Spectrometry (GC–MS) analysis was used to identify the volatile compounds. The volatile compounds were determined on a GC‐2030 gas chromatograph equipped with a QP2020 NX mass selective detector (MS) (Shimadzu). A fused‐silica capillary column Rtx‐Wax (60 m × 0.25 mm × 0.25 μm, Restek, USA) was used for analysis. The flow rate of helium was 1 mL/min. Analysis was conducted in splitless mode with an injection temperature of 250°C. The sampling time was 3 min. The oven temperature was held at 40°C for 6 min, then increased from 40°Cto 160°C at 3°C/min, and finally increased from 160°C to 230°C at 10°C/min to be held for 5 min at 230°C. The mass spectrometer operated in electron impact mode (EI) at a voltage of 70 eV and the MS scanning was from 40 to 206 amu. Aroma compounds were identified by comparing their mass spectra with the mass spectra of libraries (Nist20, Wiley 7.0). The calibration of aroma compounds was made as described previously (Bureš et al. 2024).

### 2.14 Statistical Analysis

The obtained dataset was analyzed using IBM SPSS Statistics 22 and XLSTAT add‐on for Microsoft Office 2016. The data are represented as means with standard deviations obtained from measurements of triplicates. A repeated measures ANOVA (analysis of variance) was used. The significance (*p* < 0.05) for chemical analyses was established using the post hoc t‐tests with Bonferroni adjustment. For pomological analysis, Tukey's HSD test was used. Pearson correlation analysis was also performed using the same statistical package. Relative change (Rc%) compared to the control samples (uncoated) was calculated by Equation (3):

(3) $$\text{Relative change}\;\left(\%\right)=\frac{a-b}{b}\times 100$$

where *a* is treated, and *b* untreated (control) sample.

## 3 Results and Discussion

### 3.1 Fruit Weight Loss and Decay Rate, Total Soluble Solids and Titratable Acidity, Visual Observation Over Time, Fruit Gloss, and CIE Color Variables

Throughout the storage period, fruit loses weight primarily due to transpiration through stomata and direct evaporation of water vapor through epidermal cells on the surface of fruits (Moggia et al. 2017). In this research, significant differences in weight loss during the common store of mandarins were observed in comparison with controls when either Chitosan or Chitosan/Phe coating was applied (Table 1). Significant differences were noted after the 5th and 9th day of common store, but on the 12th day, only the Chitosan/Phe treatment remained significantly different compared to control samples. Weight loss during cold storage, however, did not show significant differences on either the 7th or the 35th day. These results align with previous studies that reported no significant changes in weight losses observed during cold storage in a high‐humidity environment (90%) for 28 days (Jurić et al. 2023). Similar findings were also reported by Ruan et al. (2022), who found that chitosan coating preserved mandarin weight in an environment with an air temperature of 23°C ± 2°C and 70% ± 5% relative humidity (RH). Xu et al. (2018) reported minimal weight loss for tangerines, but it should be noted that the fruits were packaged separately in polyethylene bags and stored at around 10°C with an RH of around 70%.

**TABLE 1:** Effect of treatments on mandarins stored at room temperature and in cold storage in terms of weight loss, share of pulp, total soluble solids and titratable acidity.

	Common store				Cold storage	
	Day 0	Day 5	Day 9	Day 12	Day 7	Day 35
Weight loss (%)						
Control		10.71 ± 4.37^a^	12.29 ± 6.63^a^	13.24 ± 2.25^a^	4.39 ± 0.82^a^	14.27 ± 2.54^a^
Phe		10.84 ± 4.38^a^	11.34 ± 5.81^a^	13.65 ± 1.79^a^	4.29 ± 0.63^a^	14.40 ± 2.13^a^
Chitosan		8.54 ± 1.68^b^	9.02 ± 3.65^b^	13.04 ± 3.09^ab^	4.19 ± 1.15^a^	14.27 ± 2.54^a^
Chitosan/Phe		8.24 ± 1.66^b^	9.03 ± 3.31^b^	11.61 ± 2.61^b^	4.21 ± 0.83^a^	13.96 ± 1.72^a^
ANOVA		< 0.0001***	0.0004**	0.1112^n.s.^	0.9229^n.s.^	0.9853^n.s.^
Share of pulp (%)						
Control	78.55 ± 2.92	81.50 ± 2.18^a^	84.82 ± 2.74^a^	87.47 ± 2.07^a^	81.40 ± 3.00^a^	83.42 ± 1.80^a^
Phe		82.65 ± 2.30^a^	82.98 ± 2.54^a^	87.28 ± 2.47^a^	79.77 ± 2.36^a^	82.45 ± 1.75^a^
Chitosan		83.11 ± 2.29^a^	83.70 ± 2.95^a^	87.89 ± 1.88^a^	79.23 ± 1,84^a^	83.29 ± 1.80^a^
Chitosan/Phe		82.76 ± 2.00^a^	84.27 ± 2.27^a^	85.83 ± 2.00^a^	79.77 ± 3.28^a^	81.72 ± 2.06^a^
ANOVA		0.2041^n.s.^	0.2710^n.s.^	0.0522^n.s.^	0.1486^n.s.^	0.1821^n.s.^
Total soluble solids (%)						
Control	11.10 ± 0.98	11.50 ± 1.06^a^	12.09 ± 1.13^a^	10.90 ± 1.35^a^	11.44 ± 0.82^a^	11.91 ± 1.18^a^
Phe		11.96 ± 1.31^a^	11.94 ± 0.98^a^	11.58 ± 1.19^a^	11.19 ± 1.34^a^	11.73 ± 0.93^a^
Chitosan		11.53 ± 0.94^a^	12.13 ± 0.71^a^	11.76 ± 1.11^a^	11.58 ± 0.91^a^	11.29 ± 0.80^a^
Chitosan/Phe		11.29 ± 1.07^a^	11.22 ± 1.07^a^	11.98 ± 0.74^a^	11.19 ± 1.03^a^	11.89 ± 1.28^a^
ANOVA		0.4191	0.04840*	0.0758^n.s.^	0.6803^n.s.^	0.4745^n.s.^
Titratable acidity (%)						
Control	0.83 ± 0.12	0.86 ± 0.17^a^	0.75 ± 0.15^a^	0.75 ± 0.17^a^	0.80 ± 0.11^a^	0.74 ± 0.13^a^
Phe		0.84 ± 0.20^a^	0.84 ± 0.22^a^	0.69 ± 0.14^a^	0.78 ± 0.18^a^	0.75 ± 0.18^a^
Chitosan		0.89 ± 0.15^a^	0.78 ± 0.13^a^	0.79 ± 0.13^a^	0.86 ± 0.14^a^	0.77 ± 0.15^a^
Chitosan/Phe		0.82 ± 0.17^a^	0.74 ± 0.12^a^	0.76 ± 0.17^a^	0.82 ± 0.14^a^	0.79 ± 0.12^a^
ANOVA		0.7184^n.s.^	0.3197^n.s.^	0.3549^n.s.^	0.4838^n.s.^	0.8886^n.s.^
TSS_TA						
Control	13.59 ± 1.64	14.04 ± 3.58	16.54 ± 3.01	15.13 ± 3.48	14.49 ± 2.20	16.30 ± 1.90
Phe		13.18 ± 2.03	15.90 ± 2.46	15.31 ± 2.52	13.83 ± 2.54	15.20 ± 3.55
Chitosan		14.91 ± 3.86	14.97 ± 3.52	17.41 ± 3.22	14.81 ± 2.25	16.41 ± 3.32
Chitosan/Phe		14.18 ± 2.61	15.44 ± 2.26	16.41 ± 3.95	14.05 ± 2.44	15.23 ± 2.33
ANOVA		0.5212^n.s.^	0.4822^n.s.^	0.2554^n.s.^	0.6742^n.s.^	0.6526^n.s.^

*Note:* Values superscripted with the same letter within a column (and according to the relative storage type and method) are not significantly different according to Tukey's HSD test (*p* < 0.05). Rc% represents a relative change (%) relative to the control sample. For ANOVA ^n.s.^, *, **, ***, nonsignificant, or significant at *p* ≤ 0.05, ≤ 0.001, or ≤ 0.0001, respectively.

**FIGURE 1:**
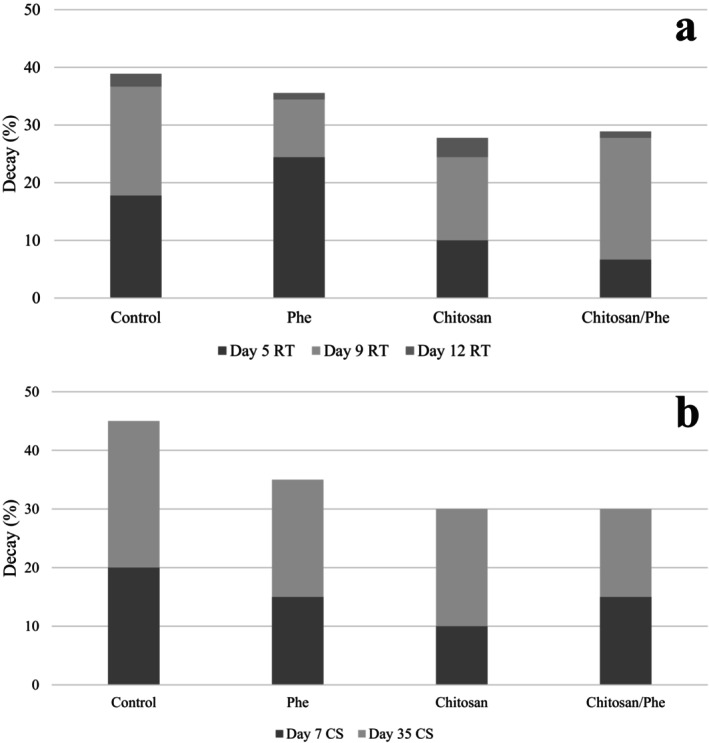
The decay rate of mandarin fruits at (a) room temperature and in (b) cold storage over time.

Regarding decay rate, Chitosan and Chitosan/Phe coatings showed positive effects during early shelf life and cold storage (Figure 1). These findings are consistent with Xu et al. (2018), who also observed a reduced decay rate for tangerines treated with chitosan coating during the first 7 days of storage (10°C, RH 70%). However, after 7 days, they found that the decay rate increased rapidly, even surpassing the decay rate of control samples. Ruan et al. (2022) similarly reported that chitosan coatings reduced the decay rate, with a maximum rate of approximately 13%, although at a much lower decay rate compared to the results of this study.

No significant differences were observed in the share of pulp, TSS, TA and TSS_TA between treatments in both storage conditions (Table 1). These parameters, which are critical for mandarin harvest prediction, remained consistent with established thresholds for TSS, TA, and TSS/TA. For example, in Israel, a TSS above 9%, TA below 1.3% and TSS_TA above 7 are the required thresholds (Tietel, Weiss, et al. 2010), while in Europe, a TSS_TA above 6.5% and juice content above 33% are necessary (UNECE 2009). Moreover, Kader (1999) states that for satisfactory mandarin fruit taste, TSS_TA values should be above 8 (Kader 1999). In the present study, all aforementioned values (Table 1) were in accordance with the discussed thresholds, confirming that mandarins were harvested at a satisfactory maturity stage (TSS_TA 13.59 at Day 0). The absence of significant differences compared to the controls during storage may be attributed either to the limited effect of the treatments or to variations in fruit maturity at harvest. Unshiu mandarins do not ripen uniformly, so harvesting is typically done in multiple stages, often spread across three intervals of 5–6 days each (Bakarić 1983). Since all the mentioned parameters (juice content, TSS, TA, TSS_TA) are determined through destructive methods, they cannot be used to assess fruit maturity uniformly across the batch. Among non‐destructive parameters, fruit color can be utilized to some extent, but its reliability is limited. Bakarić (1983) notes that Satsuma mandarin fruit is considered mature when its peel color begins changing from green to yellow‐orange, with 1/3 to 2/3 of the surface showing the yellow‐orange hue. However, Satsuma mandarins often exhibit significant variability in coloration, with internal quality indicators for commercial maturity being reached before the external coloration change is fully complete (Lado et al. 2014). Similarly, Goldenberg et al. (2017) states that early in the harvest season, mandarins may still have a green peel, even though they have reached internal maturity. This makes it challenging to assess “Owari satsuma” maturity based solely on peel coloration, leading to variations in fruit maturity that can affect fruit quality traits. In this study, only mandarins with 100% yellow‐orange peel coloration were selected, and others were discarded to ensure, as much as possible, a uniform maturity level.

Similar results for TSS and TA were reported by Arnon et al. (2014) for two types of mandarins, oranges, and grapefruits with bilayer edible coatings. Likewise, layer‐by‐layer (LbL) coatings and commercial wax coatings had no significant effects on the TTS and TA levels in mandarin fruit juice (Arnon‐Rips and Poverenov 2018). Similar results were observed for strawberries, where chitosan or chitosan LbL coatings had little to no effect on the TSS and TA after 8 days of storage at 0°C (Yan et al. 2019).

**FIGURE 2:**
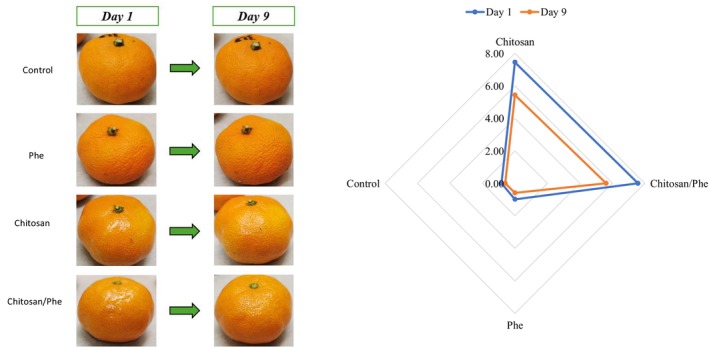
Mandarin fruits visual change over time (initial and after 1 and 9 days) stored at room temperature (20°C ± 2°C, 60% ± 10% relative humidity). Mandarin fruit gloss was evaluated on a 0–10 scale (0 = no gloss; 10 = very glossy) in a paired comparison blind test: Control, Phe, Chitosan, and Chitosan/Phe.

Regarding sensory quality, no adverse effects were observed in terms of stickiness, peeling, or curling initially or throughout storage. Chitosan and Chitosan/Phe coatings improved the glossiness of mandarin fruits (Figure 2). A small decline in fruit gloss was observed after 9 days, which was confirmed by sensory analysis. Mandarins coated with Chitosan and Chitosan/Phe were rated significantly higher in gloss and aesthetic appeal compared to control samples on days 1 and 9 of storage at room temperature. Moreover, it has to be noted that consumers found coated mandarins more desirable and would prefer choosing aesthetically more pleasant fruit. Previous research also highlighted that chitosan‐based layer‐by‐layer coatings (Jurić et al. 2023) enhance fruit glossiness, making mandarins more visually appealing to consumers (Arnon et al. 2015). Nevertheless, it is important to ensure that all ingredients used for fruit coating have minimal impact on sensory quality while it can be desirable to have improved glossiness or coloration since this can make fruit more attractive to the consumer (Jurić et al. 2024; Kumar, Khan, et al. 2024).

### 3.2 Total Protein Content, Total Polyphenolic Content, Total Flavonoid Content and Antioxidant Activity

The investigation into the protein content of mandarin fruits under various storage treatments revealed significant differences (Table 2). At room temperature, the Chitosan and Chitosan/Phe treatments resulted in mean relative changes of 8.72% and 10.74%, respectively. During cold storage, the protein content showed relative changes of 8.78% (Phe), 8.11% (Chitosan), and 9.46% (Chitosan/Phe). The decline in protein content over time is likely due to the collapse of cell membranes, protein leakage, and the reduction of carbohydrates, as insoluble proteins from the cell membrane attach to the cell wall and are used for cellular respiration as a source of carbon and nitrogen (Perdones et al. 2012; Mantilla et al. 2013). Chitosan/Phe contributes to protein content stabilization in mandarins during storage by mitigating protein degradation. The protective effect of Chitosan/Phe could be attributed to its ability to form a semi‐permeable coating that reduces oxidative stress and enzymatic degradation. Chitosan has been recognized for its ability to regulate metabolic pathways, thereby contributing to protein stability by reducing membrane damage and preventing protein leakage. Additionally, small molecules such as phenylalanine can function as protein stabilizers, further enhancing structural integrity and preserving protein functionality (Adibi et al. 2024).

**TABLE 2:** Total protein content in mandarins throughout the storage time under the two storage conditions.

Total protein content (g bovine serum albumin/100 g f.w.)						
Common store						
	Day 0	Day 5	Day 9	Day 12	Mean	Mean Rc%
Control	1.56 ± 0.06	1.59 ± 0.03^a^	1.60 ± 0.09^a^	1.30 ± 0.04^a^	1.49 ± 0.15^a^	
Phe		1.69 ± 0.20^ab^	1.58 ± 0.10^a^	1.29 ± 0.06^a^	1.52 ± 0.21^ab^	2.01
Chitosan		1.71 ± 0.14^ab^	1.64 ± 0.08^a^	1.52 ± 0.06^b^	1.62 ± 0.13^bc^	8.72
Chitosan/Phe		1.75 ± 0.09^b^	1.63 ± 0.08^a^	1.57 ± 0.07^b^	1.65 ± 0.11^c^	10.74
ANOVA		0.2616^n.s.^	0.7067^n.s^	< 0.0001***	0.0095*	

Cold storage						
	Day 0	Day 7	Day 35		Mean	Mean Rc%
Control	1.56 ± 0.06	1.46 ± 0.07^a^	1.52 ± 0.10^a^		1.48 ± 0.09^a^	
Phe		1.57 ± 0.06^ab^	1.65 ± 0.09^b^		1.61 ± 0.09^b^	8.78
Chitosan		1.56 ± 0.10^b^	1.61 ± 0.09^b^		1.60 ± 0.09^b^	8.11
Chitosan/Phe		1.62 ± 0.03^b^	1.62 ± 0.09^b^		1.62 ± 0.06^b^	9.46
ANOVA		0.0011*	0.0114*		< 0.0001***	

*Note:* Values superscripted with the same letter within a column (and according to the relative storage type and method) are not significantly different according to the post hoc *t*‐test (*p* < 0.05). Rc% represents a relative change (%) relative to the control sample. For ANOVA ^n.s.^, *, ***, nonsignificant, or significant at *p* ≤ 0.05, ≤ 0.001, or ≤ 0.0001, respectively.

The results of TPC (Table 3) were comparable to other Sats‐uma mandarin genotypes, with values ranging from 3.50 to 22.53 mg of gallic acid equivalent per 100 g (Yazici et al. 2023). Polyphenolic content varied significantly between treatments during both room temperature and cold storage. Treatments, particularly Chitosan and Chitosan/Phe, stimulated polyphenolic synthesis. For example, at room temperature, all treatments resulted in higher average values of TPC compared to the control samples. Overall, Chitosan and Chitosan/Phe treatments on days 9 and 12 resulted in significantly higher polyphenolic content than the control, especially on day 12, where Chitosan/Phe reached 47.31 mg GAE/100 g. When considering the relative change over time, the Chitosan/Phe treatment showed a 28.47% increase in TPC compared to the control. During cold storage, TPC increased significantly with Phe, Chitosan and Chitosan/Phe treatments, showing average increases of 16.19%, 23.46% and 42.16%, respectively, over the control sample. This highlights the effectiveness of these coatings, particularly Chitosan/Phe, in preserving polyphenolics over extended cold storage periods.

**TABLE 3:** Changes in total polyphenolic and total flavonoid content and antioxidant activity (DPPH and ABTS methods) in mandarins throughout the storage time under the two storage conditions.

	Common store						Cold storage			
	Day 0	Day 5	Day 9	Day 12	Mean	Rc%	Day 7	Day 35	Mean	Rc%
Total polyphenolic content (mg GAE/100 g f.w.)										
Control	32.99 ± 2.24	36.09 ± 1.79^a^	29.04 ± 2.94^a^	30.12 ± 1.25^a^	32.04 ± 3.67^a^		28.42 ± 1.44^a^	22.26 ± 0.99^a^	25.34 ± 3.32^a^	
Phe		39.89 ± 2.31^a^	33.92 ± 2.40^ac^	35.99 ± 1.51^b^	36.19 ± 3.12^b^	12.95	32.03 ± 2.23^bc^	26.85 ± 1.50^b^	29.44 ± 3.22^b^	16.19
Chitosan		37.58 ± 2.80^a^	39.04 ± 1.77^b^	44.56 ± 1.51^c^	40.39 ± 3.67^c^	26.08	31.20 ± 1.56^b^	31.37 ± 1.79^b^	31.28 ± 1.68^b^	23.46
Chitosan/P he		40.27 ± 3.77^a^	35.75 ± 1.33^c^	47.31 ± 2.15^c^	41.16 ± 5.51^c^	28.47	33.48 ± 1.47^c^	39.84 ± 1.65^c^	36.02 ± 3.48^c^	42.16
ANOVA		< 0.0001***	< 0.0001***	< 0.0001***	< 0.0001***		0.0012*	< 0.0001***	< 0.0001***	
Total flavonoid content (mg QE/100 g f.w.)										
Control	16.63 ± 0.48	17.97 ± 0.36^a^	17.68 ± 1.24^a^	18.37 ± 0.52^a^	18.01 ± 0.85^a^		15.14 ± 0.88^a^	15.51 ± 0.37^a^	15.29 ± 0.75^a^	
Phe		17.59 ± 2.25^a^	17.71 ± 1.28^a^	20.67 ± 1.33^b^	18.65 ± 2.20^a^	3.55	16.88 ± 1.73^ab^	18.28 ± 0.30^b^	17.58 ± 1.43^b^	14.98
Chitosan		18.51 ± 1.49^a^	19.35 ± 0.43^b^	23.58 ± 0.78^c^	20.48 ± 2.43^b^	13.71	15.53 ± 0.51^a^	16.47 ± 1.00^ab^	16.00 ± 0.92^a^	4.64
Chitosan/Phe		19.62 ± 2.08^a^	18.95 ± 0.86^ab^	26.23 ± 1.39^d^	21.60 ± 3.62^b^	19.93	18.28 ± 0.99^b^	16.82 ± 0.25^b^	17.70 ± 1.06^b^	15.76
ANOVA		0.2930 ^n.s.^	0.0326*	< 0.0001**	0.0002**		0.0001***	< 0.0001***	< 0.0001***	
DPPH (μmol TE/100 g f.w.)										
Control	145.98 ± 4.61	158.59 ± 5.04^a^	156.76 ± 4.38^a^	141.53 ± 7.27^a^	152.29 ± 9.54^a^		105.38 ± 9.97^a^	129.63 ± 4.10^a^	117.50 ± 14.32^a^	
Phe		163.09 ± 17.10^a^	155.98 ± 13.92^ac^	159.47 ± 7.06^b^	159.51 ± 13.67^a^	4.74	118.44 ± 5.35^b^	137.80 ± 6.76^a^	128.12 ± 11.44^ab^	9.04
Chitosan		166.12 ± 6.44^a^	174.02 ± 4.55^b^	176.09 ± 3.68^c^	172.08 ± 6.61^b^	12.99	119.59 ± 5.10^b^	148.87 ± 6.94^b^	134.23 ± 15.86^b^	14.24
Chitosan/Phe		168.64 ± 14.20^a^	163.81 ± 1.38^c^	179.70 ± 8.71^c^	170.72 ± 11.72^b^	12.10	119.69 ± 9.41^b^	160.36 ± 4.27^c^	135.95 ± 21.39^b^	15.70
ANOVA		0.5822^n.s.^	0.0046*	< 0.0001***	< 0.0001***		0.0223*	< 0.0001***	0.0465*	
ABTS (μmol TE/100 g f.w.)										
Control	190.14 ± 3.13	192.59 ± 12.64^a^	183.71 ± 14.42^a^	206.74 ± 5.61^a^	194.35 ± 14.93^a^		142.66 ± 10.35^a^	192.03 ± 10.51^a^	167.34 ± 26.80^a^	
Phe		193.02 ± 17.87^a^	203.33 ± 10.34^b^	227.00 ± 10.67^a^	207.79 ± 19.56^b^	6.92	158.43 ± 10.40^b^	201.33 ± 4.86^ab^	179.88 ± 22.93^ab^	7.49
Chitosan		192.90 ± 7.99^a^	220.55 ± 5.06^c^	248.65 ± 14.91^b^	220.70 ± 24.94^b^	13.56	171.97 ± 9.04^bc^	211.53 ± 10.33^b^	191.75 ± 22.03^b^	14.59
Chitosan/Phe		190.61 ± 21.69^a^	210.22 ± 4.53^c^	262.37 ± 20.73^b^	221.07 ± 34.98^b^	13.75	173.64 ± 7.75^c^	232.59 ± 6.99^c^	197.22 ± 29.82^b^	17.86
ANOVA		0.9944^n.s.^	< 0.0001***	< 0.0001***	0.0064*		0.0001***	0.0001***	0.0468*	

*Note:* Values superscripted with the same letter within a column (and according to the relative storage type and method) are not significantly different according to the post hoc *t*‐test (*p* < 0.05). Rc% represents a relative change (%) relative to the control sample (mean values). For ANOVA ^n.s.^, *, **, nonsignificant, or significant at *p* ≤ 0.05, ≤ 0.001, or ≤ 0.0001, respectively.

Phe plays a key role in stimulating the phenylpropanoid pathway, which leads to increased polyphenolic compound synthesis in mandarins over time. The phenylpropanoid pathway drives numerous metabolic reactions in plants, and exogenous Phe has been shown to activate related pathways and antioxidant enzymes. The pathway begins with Phe, catalyzed by PAL, which is responsible for producing various metabolites, such as phenolics and flavonoids, in response to stress or injury (Patel, Fanyuk, et al. 2023; Patel, Maurer, et al. 2023). Additionally, the acidic environment created by dissolving chitosan in acid can stimulate flavonoid synthesis (Jurić et al. 2023).

Similar to polyphenolics, total flavonoid content (TFC) also increased with Chitosan and Chitosan/Phe coatings (Table 3), with clear differences observed during extended cold storage (up to 35 days). By Day 12 at room temperature, Chitosan/Phe treatment resulted in the highest flavonoid content at 26.23 mg QE/100 g, the highest amount across the various treatments. Similar trends were detected in clementines with TFC ranging from 18.57 to 19.87 mg/100 g of f.w. sample (De Ancos et al. 2017). In cold storage, Phe and Chitosan/Phe treatments yielded the highest TFC, significantly differing from both the control sample and Chitosan treatment. This indicates the role of exogenous Phe in enhancing flavonoid synthesis by increasing precursor availability. Flavonoids are synthesized through the phenylpropanoid pathway, where Phe is converted into 4‐coumaroyl‐CoA, which subsequently enters the flavonoid biosynthesis pathway. The first enzyme in this process, chalcone synthase, produces chalcone scaffolds, the foundation for all flavonoids (Falcone Ferreyra et al. 2012). Yan et al. (2019) demonstrated from a metabolomic perspective that chitosan coating enhanced flavonoid synthesis in strawberries during cold storage.

Polyphenols and flavonoids are known for their strong antioxidant properties (Lee and Kim 2022). Enhancing their synthesis through Phe application, may not only improve the nutritional quality of mandarins but also extend their shelf life by better combating oxidative stress during storage. The increase in flavonoids could be partially explained by the stimulation caused by the acidic environment from the chitosan coating (Jurić et al. 2023). Additionally, (Patel, Fanyuk, et al. 2023; Patel, Maurer, et al. 2023) found that postharvest Phe treatment in mangoes reduced chilling injuries by inducing genes related to plant‐pathogen interactions, plant hormone signal transduction, and the phenylpropanoid pathway, leading to increased levels of flavonoids like quercetin and kaempferol glycosides, anthocyanins, and antioxidants.

The antioxidant activity, measured by DPPH and ABTS assays, closely followed the trends in TPC and TFC (Table 3). Chitosan and Chitosan/Phe treatments maintained higher antioxidant activity over time. By Day 12 at room temperature, Chitosan/Phe reached 179.70 μmol TE/100 g, indicating that these coatings help enhance and sustain high antioxidant levels. Similar levels were previously reported (De Ancos et al. 2017), with a range from 343.69 to 371.88 μmol AA/100 g (f.w.). ABTS assay results mirrored those of DPPH, with Chitosan and Chitosan/Phe consistently showing superior antioxidant activity over the storage period. Comparable levels were also found by De Ancos et al. (2017), with a range from 357.36 to 402.45 μmol AA/100 g (f.w.).

Under cold storage conditions, ABTS values for Chitosan/Phe further increased to 232.59 μmol TE/100 g by day 35, highlighting its effectiveness in preserving antioxidant properties over the long term. Similar results have been reported in previous studies by Candir et al. (2018) and Reyes‐Avalos et al. (2019), where Chitosan coating was the most effective treatment in enhancing both polyphenolic content and antioxidant activity in pomegranate fruit and figs, respectively.

### 3.3 Flavanones

When examining specific flavanones such as narirutin and hesperidin, which are among the most abundant flavanones in mandarins, interesting outcomes were observed (Table 4). The narirutin and hesperidin content in the pulp of mandarins found in this study aligns with other research by Xu et al. (2009) and Maslov Bandić et al. (2023). At the end of shelf life, mandarins treated with Phe, and Chitosan/Phe had significantly higher concentrations of narirutin relative to the control and Chitosan. After 35 days of cold storage content was significantly higher in mandarins treated with Chitosan and Chitosan/Phe with the latter being significantly higher than the former. At the end of shelf life significantly higher content of hesperidin was observed in Phe, Chitosan and Chitosan/Phe than in control. At the end of cold storage significantly the highest content was in mandarins treated with Chitosan/Phe.

**TABLE 4:** Changes in narirutin and hesperidin content of mandarins throughout the storage time under the two storage conditions.

	Narirutin (mg/g d.w.)			Hesperidin (mg/g d.w.)		
	Day 0	Common store Day 12	Cold storage Day 35	Day 0	Common store Day 12	Cold storage Day 35
Control	5.84 ± 0.06	7.48 ± 0.07^a^	6.62 ± 0.10^a^	13.39 ± 0.34	15.07 ± 0.18^a^	12.92 ± 0.19^a^
Phe		8.14 ± 0.04^b^	6.95 ± 0.08^a^		16.04 ± 0.10^b^	14.68 ± 0.28^b^
Chitosan		7.42 ± 0.27^a^	7.52 ± 0.13^b^		17.04 ± 0.04^c^	14.90 ± 0.30^b^
Chitosan/Phe		9.09 ± 0.01^c^	8.27 ± 0.09^c^		18.32 ± 0.11^d^	16.52 ± 0.31^c^
ANOVA		0.0002**	< 0.0001***		< 0.0001***	0.0044*

*Note:* Values superscripted with the same letter within a column (and according to the relative storage type and method) are not significantly different according to the post hoc *t*‐test (*p* < 0.05). For ANOVA ^n.s.^, *, **, ***, nonsignificant, or significant at *p* ≤ 0.05, ≤ 0.001, or ≤ 0.0001, respectively.

### 3.4 Syneprhine Content

The changes in synephrine content across the different treatments and storage conditions can be observed in Table 5. The data indicate that temperature influences synephrine accumulation. At room temperature, synephrine levels increased over time, particularly in Chitosan and Chitosan/Phe‐treated mandarins, with relative changes of 26.60% and 26.86%, respectively, compared to the control. Conversely, under cold storage conditions, synephrine levels showed less pronounced changes, with Chitosan/Phe treatment leading to the highest relative increase (18.83%). These findings suggest that elevated storage temperatures may promote synephrine accumulation, possibly due to enhanced metabolic activity and stress responses in the fruit. Meanwhile, cold storage appears to slow this accumulation, likely by reducing enzymatic activity and metabolic processes. The results highlight the potential of Chitosan‐based coatings not only to improve the nutritional quality of mandarins but also to enhance their commercial value, as elevated synephrine levels are associated with health benefits (Ruiz‐Moreno et al. 2021; Shara et al. 2018).

**TABLE 5:** Synephrine content in mandarins throughout the storage time under the two storage conditions.

Synephrine (g/100 g f.w.)						
Common store						
	Day 0	Day 5	Day 9	Day 12	Mean	Mean Rc%
Control	3.38 ± 0.25	3.51 ± 0.42^a^	3.89 ± 0.06^a^	3.91 ± 0.18^a^	3.76 ± 0.33^a^	
Phe		3.59 ± 0.47^a^	3.75 ± 0.40^a^	3.97 ± 0.21^a^	3.77 ± 0.41^a^	0.27
Chitosan		4.18 ± 0.51^b^	4.71 ± 0.08^b^	5.71 ± 0.08^b^	4.76 ± 0.67^b^	26.60
Chitosan/Phe		4.61 ± 0.54^b^	4.52 ± 0.21^b^	5.40 ± 0.02^b^	4.77 ± 0.51^b^	26.86
ANOVA		0.1569^n.s.^	0.0078*	0.0005*	0.0001**	

Cold storage						
	Day 0	Day 7	Day 35		Mean	Mean Rc%
Control	3.38 ± 0.25	2.92 ± 0.12^a^	3.33 ± 0.34^a^		3.03 ± 0.26^a^	
Phe		3.44 ± 0.12^b^	3.40 ± 0.19^a^		3.42 ± 0.35^ab^	11.04
Chitosan		3.43 ± 0.10^b^	3.74 ± 0.01^ab^		3.56 ± 0.15^b^	15.58
Chitosan/Phe		3.45 ± 0.23^b^	3.97 ± 0.20^b^		3.66 ± 0.30^b^	18.83
ANOVA		0.0491*	0.5916^n.s.^		0.0494*	

*Note:* Values superscripted with the same letter within a column (and according to the relative storage type and method) are not significantly different according to the post hoc *t*‐test (*p* < 0.05). Rc% represents a relative change (%) relative to the control sample. For ANOVA ^n.s.^, *, ** nonsignificant, or significant at *p* ≤ 0.05, ≤ 0.001, or ≤ 0.0001, respectively.

### 3.5 Organic Acids

The primary reason for the degradation in taste quality of citrus fruit during postharvest storage is the reduction in TA. Delayed fruit senescence is typically associated with a high concentration of organic acids, which are critical for maintaining fruit quality during storage (Angioni and Schirra 2011; Sun et al. 2013). Appropriate storage temperatures can slow metabolic activities, particularly the rate of respiration, and extend the shelf life of fruit while preserving acceptable quality features (Hussain et al. 2017). Organic acid content plays a key role in preserving fruit flavor and regulating fruit senescence. There is often a negative correlation between fruit weight loss and changes in organic acid content, affecting both flavor and storage performance (Sheng et al. 2017). Edible coatings, such as Chitosan, are often used to mitigate postharvest losses and preserve quality.

Oxalic acid, a critical component in regulating plant growth and stress responses, including defense against biotic and abiotic factors, is known for its strong acidity and chelating ability (Li et al. 2022). At room temperature, all treatments increased oxalic acid levels by the 12th day compared to control, with Chitosan and Chitosan/Phe showing the highest concentrations of 10.15 and 9.55 mg/100 g, respectively (Table 6). Although this increase was less pronounced during cold storage, Chitosan/Phe treatment still demonstrated an increase to 9.14 mg/100 g by the 35th day, indicating its effectiveness in modifying the organic acid profile even at lower temperatures. Oxalic acid's role in inhibiting ethylene biosynthesis, delaying ripening, and prolonging storage periods is significant for controlling browning reactions and maintaining fruit quality (Eroğul et al. 2023). It also reacts against environmental stress factors and affects the biochemical and quality parameters of fruits (Liang et al. 2009). Herein, it can be speculated that the treatments may affect the activity of enzymes involved in oxalate biosynthesis or its degradation pathways. For instance, the treatments could modulate the expression or activity of glycolate oxidase, or impact the ascorbic acid pathway in distinct ways, thereby leading to the changes in oxalic acid levels observed in this study. While oxalic acid metabolism has been extensively studied in microorganisms, fungi, and animals, its regulation in plants remains less understood (Li et al. 2022).

**TABLE 6:** Changes in organic acid (oxalic, malic, ascorbic and citric) composition in mandarins throughout the storage time under the two storage conditions.

	Common store						Cold storage			
	Day 0	Day 5	Day 9	Day 12	Mean	Rc%	Day 7	Day 35	Mean	Rc%
Oxalic acid (mg/100 g f.w.).										
Control	7.31 ± 0.18	7.31 ± 1.04^a^	9.18 ± 0.25^a^	8.33 ± 0.36^a^	8.27 ± 1.01^a^		6.71 ± 0.43^a^	8.02 ± 0.70^a^	7.36 ± 0.88^a^	
Phe		7.64 ± 0.66^a^	8.95 ± 1.16^a^	9.25 ± 0.48^ab^	8.61 ± 1.08^ab^	4.11	6.92 ± 0.40^a^	7.70 ± 0.21^a^	7.31 ± 0.51^a^	−0.68
Chitosan		8.51 ± 0.66^a^	9.81 ± 0.22^a^	10.15 ± 0.55^b^	9.49 ± 0.87^b^	14.75	7.25 ± 0.45^a^	7.58 ± 0.65^a^	7.42 ± 0.58^a^	0.82
Chitosan/Phe		9.31 ± 0.28^a^	9.19 ± 0.06^a^	9.55 ± 0.23^b^	9.35 ± 0.26^b^	13.06	7.76 ± 0.61^a^	9.14 ± 0.15^a^	8.31 ± 0.83^a^	12.91
ANOVA		0.0842^n.s.^	0.5544^n.s.^	0.0159*	0.0231*		0.2228^n.s.^	0.1247^n.s.^	0.1599^n.s.^	
Malic acid (mg/100 g f.w.).										
Control	41.49 ± 3.51	49.36 ± 1.74^a^	51.13 ± 3.57^a^	44.17 ± 2.09^a^	48.08 ± 4.10^a^		47.29 ± 2.18^a^	27.81 ± 5.39^a^	37.55 ± 10.57^a^	
Phe		46.54 ± 2.83^a^	51.63 ± 1.16^a^	42.66 ± 3.20^a^	46.94 ± 4.48^ab^	−2.37	49.75 ± 1.74^a^	39.78 ± 3.27^a^	44.77 ± 5.63^a^	19.23
Chitosan		41.54 ± 2.92^a^	40.83 ± 5.40^ab^	44.62 ± 2.79^a^	42.05 ± 4.29^c^	−12.54	39.90 ± 4.98^a^	39.59 ± 1.67^b^	39.75 ± 3.72^a^	5.86
Chitosan/Phe		47.58 ± 1.51^a^	39.86 ± 1.53^b^	43.44 ± 2.44^a^	43.63 ± 3.67^bc^	−9.26	43.71 ± 1.85^a^	40.49 ± 4.38^a^	42.10 ± 3.73^a^	12.12
ANOVA		0.0838^n.s.^	0.0119*	0.9102^n.s.^	0.0320*		0.0716^n.s.^	0.0464*	0.4043^n.s.^	
Ascorbic acid (mg/100 g f.w.).										
Control	24.67 ± 2.79	19.73 ± 0.98^a^	20.00 ± 1.35^a^	18.02 ± 0.92^a^	19.25 ± 1.41^a^		16.24 ± 1.56^a^	21.90 ± 1.49^ab^	19.07 ± 3.21^a^	
Phe		20.05 ± 2.17^ab^	18.96 ± 1.83^a^	19.10 ± 1.73^a^	19.37 ± 1.98^a^	0.62	16.54 ± 1.13^a^	19.94 ± 0.53^a^	18.24 ± 1.92^a^	−4.35
Chitosan		22.78 ± 0.58^b^	22.25 ± 1.34^a^	22.95 ± 1.19^b^	22.66 ± 1.13^b^	17.71	20.50 ± 1.19^b^	20.66 ± 0.37^ab^	20.58 ± 0.89^a^	7.92
Chitosan/Phe		20.95 ± 2.43^ab^	19.57 ± 0.26^a^	20.07 ± 1.01^ab^	20.20 ± 1.63^a^	4.94	18.72 ± 1.84^a^	22.40 ± 0.55^b^	20.19 ± 2.33^a^	5.87
ANOVA		0.3536 ^n.s.^	0.1500^n.s.^	0.0217*	0.0004**		0.0597^n.s.^	0.1256^n.s.^	0.3750^n.s.^	
Citric acid (mg/100 g f.w.).										
Control	773.25 ± 8.31	736.00 ± 54.97^a^	746.47 ± 46.77^ab^	667.79 ± 29.55^a^	722.88 ± 56.60^a^		692.81 ± 73.34^a^	696.85 ± 54.01^a^	694.83 ± 64.44^a^	
Phe		701.59 ± 66.48^ab^	779.61 ± 23.86^a^	679.12 ± 27.55^a^	743.51 ± 97.53^ab^	2.85	696.49 ± 105.19^a^	744.33 ± 53.33^ab^	720.41 ± 86.76^ab^	3.68
Chitosan		889.83 ± 47.69^b^	782.41 ± 15.39^a^	794.35 ± 23.69^b^	822.19 ± 57.75^b^	13.74	784.70 ± 22.57^a^	840.85 ± 10.00^ab^	798.74 ± 31.19^b^	14.95
Chitosan/Phe		845.31 ± 34.96^ab^	724.53 ± 7.54^b^	722.93 ± 54.64^a^	764.26 ± 68.61^ab^	5.72	782.63 ± 42.38^a^	944.07 ± 76.35^b^	847.21 ± 98.31^b^	21.93
ANOVA		0.0351*	0.0084*	0.0510^n.s^	0.0606^n.s^		0.3939^n.s.^	0.0323*	0.0226*	

*Note:* Values superscripted with the same letter within a column (and according to the relative storage type and method) are not significantly different according to the post hoc *t*‐test (*p* < 0.05). Rc% represents a relative change (%) relative to the control sample (mean values). For ANOVA ^n.s.^, *, ** nonsignificant, or significant at *p* ≤ 0.05, ≤ 0.001, or ≤ 0.0001, respectively.

Malic acid, through being more abundant in apples (Wu et al. 2007) and pears (Lu et al. 2011), showed high variability across treatments in this study. A significant reduction in malic acid content was observed only in the Chitosan treatment at the end of cold storage. This variability suggests that Chitosan may inhibit certain enzymes involved in malic acid production, highlighting the complexity of organic acid metabolism in different fruits (Ma et al. 2018).

Citrus fruits, including mandarins, are a major source of ascorbic acid for human consumption (Alós et al. 2021). As can be observed from Table 6, Phe treatment did not significantly affect ascorbic acid synthesis, but Chitosan consistently maintained higher ascorbic acid levels during both shelf life and cold storage. This may be due to the low O_2_ permeability of chitosan‐based coatings, which could inhibit enzymes responsible for ascorbic acid oxidation (Dang et al. 2010; Kerch et al. 2011; Tokatlı and Demirdöven 2020).

Citric acid, the dominant major organic acid in citrus, accumulates in the juice sac cells of citrus fruit (Etienne et al. 2013; Lin et al. 2016). Interestingly, citric acid showed a remarkable increase in mandarins treated with Chitosan/Phe during cold storage. After 35 days, citric acid content reached 944 mg/100 g f.w., representing a 21.93% relative increase compared to the control. At room temperature, Chitosan treatment also led to a substantial rise in citric acid after 12 days, with Chitosan/Phe‐treated mandarins closely following, although in this case no significant difference was observed. Similar to other fruits, citric acid accumulation significantly influences the quality and flavor of the fruit at maturity. A taste that is too sour is frequently the consequence of acid content levels that exceed the required level. Conversely, fruit with a low acid content at maturity may be excessively sweet and may be linked to elevated levels of secondary metabolites, including alcohols and aldehydes, which can negatively impact fruit flavor during either the pre‐harvest or post‐harvest storage phase (Porat et al. 2002; Hussain et al. 2017).

### 3.6 Aroma

Table 7 shows the variations in the concentration of various aromatic compounds across different treatments and storage conditions. The degradation and oxidation of these volatile compounds, particularly during storage can lead to significant changes in the aroma profile of citrus fruits. Some compounds are associated with the perception of off‐flavors. For example, E‐2‐hexenal, *trans*‐2‐octenal and 1‐octen‐3‐ol are described as having greeny, nutty, fatty or mushroom‐like odor characteristics (Tietel, Plotto, et al. 2010). Decenal, described as beefy and musty, was found in fruits held at room temperature but not in cold storage, indicating that certain off‐flavors are more likely to develop at room temperatures.

**TABLE 7:** The aromatic profile of mandarins on the starting day and at the end of the shelf life at room temperature and in cold storage.

Compound	Odor desrciption	Day 0	Common store, Day 12				Cold storage, Day 35			
		Mass concentration, μg/kg mandarin fruit								
			Control	Phe	Chitosan	Chitosan/Phe	Control	Phe	Chitosan	Chitosan/Phe
Aldehydes										
Hexanal	Grass^a^	1.361	1.651	2.152	1.245	1.378	—	1.941	6.763	—
Heptanal	Fatty, citrus^a^	0.073	0.049	0.062	0.037	0.069	0.064	0.084	—	0.132
trans‐2‐Hexenal, (E)‐	Green, leaf^b^	0.682	0.971	0.480	0.176	0.508	0.745	0.655	1.426	—
Octanal	Fat, citrus, green^a^	0.071	0.044	0.054	—	0.077	0.054	0.082	0.133	0.959
trans‐2‐Heptenal	Green^a^	0.047	0.288	—	—	0.064	0.152	—	0.224	—
Nonanal	Piney, floral, citrusy^a^	0.056	0.058	0.071	0.093	0.290	0.069	0.105	—	0.105
trans‐2‐Octenal	Green, nut, fat^a^	—	0.114	—	—	0.130	—	—	—	—
Decanal	Beefy, musty, marine, cucumber^b^	0.081	0.000	0.022	0.035	0.100	0.033	—	—	—
trans‐2‐Nonenal	Floral^a^	0.029	0.019	0.024	0.055	0.025	—	0.056	0.084	0.055
trans‐2‐Decenal	Aldehyde, sweet^c^	0.036	0.018	0.025	0.067	0.049	0.027	0.042	0.040	0.080
Ketones										
6‐methyl‐5‐hepten‐2‐on	Sweet, green^a^	0.005	0.035	0.041	0.009	0.018	—	0.039	0.045	—
D‐Carvone	Mint, basil, fennel^a^	0.008	—	0.007	0.009	0.010	0.006	0.008	0.009	0.017
Alcohols										
1‐Hexanol	Resin, paper^a^	0.212	2.862	3.232	2.734	0.943	1.708	7.029	—	—
trans‐3‐Hexen‐1‐ol	Green, leaf^a^	—	0.065	0.055	0.033	0.030	0.052	0.140	0.070	—
1‐Octen‐3‐ol	mushroom^a^	—	0.014	0.018	0.018	0.009	0.011	0.027	0.036	0.041
1‐Octanol	soapy^a^	0.055	0.018	0.033	0.168	0.215	—	0.063	—	0.132
Decanol	Waxy, fruity^b^	0.025	—	0.005	0.027	—	—	—	—	0.007
Terpenes										
Alpha Pinene	Pine tree, ethereal^a^	0.396	0.077	0.224	0.172	0.151	0.120	0.899	0.455	1.305
Beta Pinene	Resinous, dry, woody^a^	—	0.023	0.112	0.146	0.135	0.109	—	0.258	0.830
β‐myrcene	Balsamic, must, spice	13.551	11.119	5.953	12.509	14.224	12.985	12.568	11.269	11.403
alpha‐ Terpinene	Lemony, citrusy^a^	0.068	—	0.035	—	—	0.024	—	—	—
Ocymene	Woody, herbal^c^	27.678	1.723	6.074	8.604	5.892	—	17.673	—	—
(E)‐beta‐Ocimene	Herbaceous, tropical, swwet^a^	0.040	—	0.037	0.060	0.042	0.031	0.045	—	—
Terpinolene	Pine, plastic^a^	0.031	—	0.028	—	0.035	0.026	0.046	—	—
trans‐Linalool Oxide	Floral^a^	0.018	0.039	0.053	0.202	0.042	0.035	0.055	0.038	0.235
Linalool	Floral, green, citrus^a^	0.178	0.005	0.003	0.603	0.119	0.005	0.004	0.001	0.004
alpha.‐Terpineol	Floral, lilac like^a^	0.067	0.014	0.017	0.360	0.223	0.012	0.020	0.029	0.116
cis‐Citral	lemin	0.012	—	0.008	0.006	0.007	—	—	—	—
Citronellol	Rose like, fresh^a^	0.016	—	—	0.020	0.018	0.009	—	0.010	—
trans‐Carveol	Caraway, solvent^a^	0.033	0.032	0.037	0.039	0.051	0.025	0.054	0.062	0.056
beta Ionone	Sweet, floral, woody^a^	0.004	0.012	0.023	0.011	0.007	0.006	0.009	0.011	0.007
Esters										
Methyl‐butanoate	Ether, sweet, fruit^a^	—	—	0.009	—	0.011	—	0.011	—	—
Geranyl acetate	Lemony^c^	0.011	0.009	0.009	0.009	0.009	—	—	—	0.009
cis‐Geranyl acetone	Fruity^c^	—	0.044	0.091	0.042	0.040	—	—	—	—

^a^ Jia et al. (2024).

^b^ Tietel, Plotto, et al. (2010); Tietel, Weiss, et al. (2010).

^c^ Obenland et al. (2013).

Interestingly, alpha‐pinene increased in cold‐stored mandarins treated with Chitosan, suggesting that this treatment may help in retaining or enhancing certain aromatic compounds. Additionally, hexanal, often associated with the fresh, green aroma of fruits, showed a higher concentration in Chitosan/Phe‐coated mandarins especially under cold storage. Comparing the two storage methods, the concentration of many volatiles like alpha‐pinene and ocimene varied, indicating the critical influence of storage conditions on the aromatic profile of treated fruits.

Phe and Chitosan/Phe treatments also promoted the formation of methyl butanoate, known for its fruity aroma. Furthermore, the combination of Chitosan/Phe generally had a positive effect on maintaining or enhancing the overall aromatic profiles compared to the control, although the impact varied across different compounds. This could indicate a synergistic effect of these treatments on the aroma profile of mandarins, helping to preserve or improve the fruit's sensory quality during storage.

## 4 Conclusions

The data obtained suggests that Chitosan and Chitosan/Phe treatments are effective in preserving and enhancing both the nutritional and sensory qualities of mandarins during postharvest storage. These treatments minimized decay, particularly in the early days of shelf life at room temperature and during cold storage, and reduced weight loss over two (Chitosan) or three (Chitosan/Phe) sampling days, which highlights their practical significance from a producer's perspective. Under common storage conditions the untreated (control) mandarins exhibited visible signs of decay prior to the ninth day of storage, indicating a rapid decline in postharvest quality under ambient conditions. In contrast, the application of the Chitosan/Phenylalanine (Chitosan/Phe) coating effectively extended the marketable shelf life to approximately 12 days, representing an increase of around 3 days compared to both the control and the Chitosan‐only treatments. Under cold storage (5°C) the control mandarins showed a notable reduction in quality before the thirty‐fifth day of storage. Conversely, fruit treated with the Chitosan/Phe coating retained acceptable physicochemical and sensory attributes throughout the entire 35‐day storage period. This result suggests that the Chitosan/Phe treatment effectively doubled the storage longevity relative to uncoated mandarins, which typically maintain acceptable quality for only 15–20 days under comparable cold storage conditions.

However, the use of Phe alone should be approached with caution due to its association with increased decay during the initial days of shelf life and its limited effect on reducing weight loss. Chitosan, especially when combined with Phe, showed a significant impact on the preservation and synthesis of bioactive compounds, including polyphenols and flavonoids, contributing to both the nutritional value and the antioxidant capacity of mandarins.

Additionally, postharvest treatments involving Phe, particularly Chitosan/Phe, influenced the aromatic profile of the fruits, preserving or enhancing desired volatiles such as alpha‐pinene and hexanal during storage. This aromatic enhancement could improve marketability and consumer acceptance by maintaining freshness and appealing aromas over time.

Future research should focus on optimizing edible coatings with varying concentrations of Phe to better understand their effects on both the preservation of bioactive compounds and sensory characteristics of mandarins, including taste and aroma. Improved polymer composition, such as by‐layer or composites, might improve the effectiveness of coating over prolonged storage by controlling the release rate of Phe. Exploring these aspects further could help refine postharvest treatment strategies to improve the overall quality and shelf life of mandarins.

## Author Contributions

**Slaven Jurić:** conceptualization (equal), data curation (equal), formal analysis (equal), investigation (equal), methodology (equal), software (equal), validation (equal), visualization (equal), writing – original draft (lead). **Marko Vuković:** conceptualization (equal), data curation (equal), formal analysis (equal), software (equal). **Marija Sigurnjak Bureš:** data curation (equal), formal analysis (equal), visualization (equal). **Katarina Sopko Stracenski:** formal analysis (equal), validation (equal). **Francesco Donsì:** supervision (equal), writing – review and editing (equal), data curation (equal). **Luna Maslov Bandić:** conceptualization, funding acquisition (equal), project administration (equal), supervision (equal), writing – review and editing (equal).

## Conflicts of Interest

The authors declare no conflicts of interest.

## Data Availability

The data that support the findings of this study are available from the corresponding author upon reasonable request.
